# Cell Death Pathways in Photodynamic Therapy of Cancer

**DOI:** 10.3390/cancers3022516

**Published:** 2011-06-03

**Authors:** Pawel Mroz, Anastasia Yaroslavsky, Gitika B Kharkwal, Michael R. Hamblin

**Affiliations:** 1 Wellman Center for Photomedicine, Massachusetts General Hospital, Boston, MA 02114, USA; E-Mails: asya8819@gmail.com (A.Y.); gkharkwal@partners.org (G.K.); hamblin@helix.mgh.harvard.edu (M.R.H.); 2 Department of Dermatology, Harvard Medical School, Boston, MA 02114, USA; 3 Boston University College of Engineering, Boston, MA 02114, USA; 4 Harvard-MIT Division of Health Sciences and Technology, Cambridge, MA 02139, USA

**Keywords:** photodynamic therapy, cell death, apoptosis, necrosis, autophagy, cancer

## Abstract

Photodynamic therapy (PDT) is an emerging cancer therapy that uses the combination of non-toxic dyes or photosensitizers (PS) and harmless visible light to produce reactive oxygen species and destroy tumors. The PS can be localized in various organelles such as mitochondria, lysosomes, endoplasmic reticulum, Golgi apparatus and plasma membranes and this sub-cellular location governs much of the signaling that occurs after PDT. There is an acute stress response that leads to changes in calcium and lipid metabolism and causes the production of cytokines and stress response mediators. Enzymes (particularly protein kinases) are activated and transcription factors are expressed. Many of the cellular responses center on mitochondria and frequently lead to induction of apoptosis by the mitochondrial pathway involving caspase activation and release of cytochrome c. Certain specific proteins (such as Bcl-2) are damaged by PDT-induced oxidation thereby increasing apoptosis, and a build-up of oxidized proteins leads to an ER-stress response that may be increased by proteasome inhibition. Autophagy plays a role in either inhibiting or enhancing cell death after PDT.

## 1. Introduction

**Figure 1.**
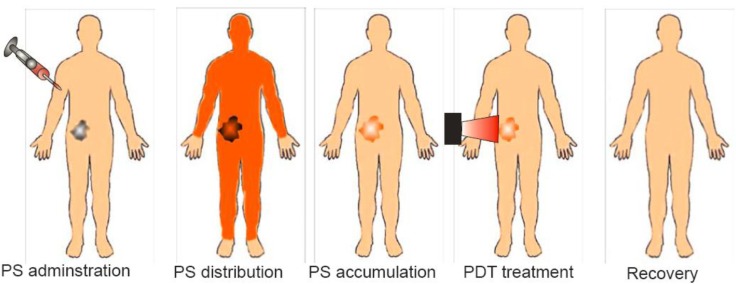
Schematic illustration of the mechanism of Photodynamic therapy (PDT). The photosensitizer (PS) is injected systemically and after sufficient time to allow its accumulation in the lesion light is delivered to produce reactive oxygen species (ROS). The tumor cells are killed by a mixture of necrosis and apoptosis, the blood supply is damaged and the host immune system activated.

Photodynamic therapy (PDT) is a promising therapeutic procedure for the management of a variety of solid tumors and non-malignant lesions. PDT is a two-step procedure that involves the administration of a photosensitizing agent [1-3], followed by activation of the drug with non-thermal light of a specific wavelength [4,5] (Figure 1). The anticancer effect of PDT is a consequence of a low-to-moderately selective degree of photosensitizer (PS) uptake by proliferating malignant cells, direct cytotoxicity of reactive oxygen species (ROS) and a severe vascular damage that impairs blood supply to the treated area [6,7]. Those biological effects of PDT are limited to the particular areas of tissues exposed to light. Additionally, PDT leads to activation of tumor directed, systemic immune responses [8-10].

**Figure 2.**
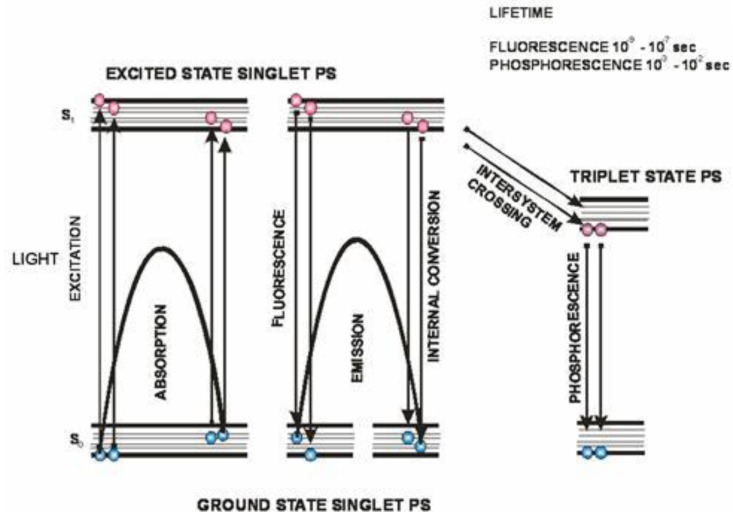
Jablonski diagram. Ground state PS absorbs a photon of correct wavelength to excite its electron to first excited singlet state that may (in addition to losing energy by heat or fluorescence emission) undergo intersystem crossing to long lived triplet state. This can undergo photochemistry by either electron transfer (Type I) or by energy transfer (Type II) to molecular oxygen to produce ROS.

**Figure 3.**
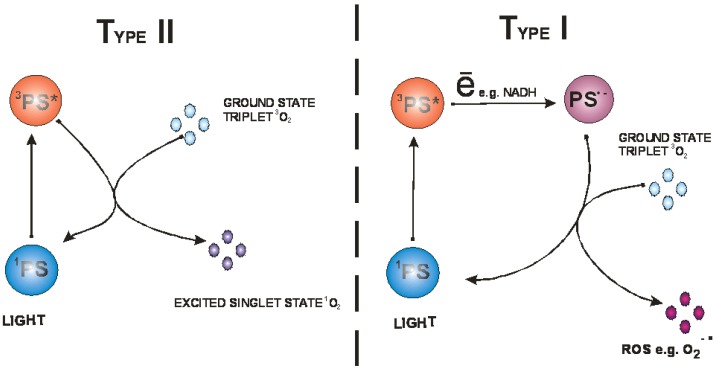
Photodynamic effect. An excited triplet state can either react directly with a reductant, e.g., an organic molecule in a cellular microenvironment, acquiring a hydrogen atom or electron to form a radical and produce a superoxide anion radical (O_2_^•-^), type I reaction or, more likely, transfer its energy to molecular oxygen (^3^O_2_) and form singlet oxygen (^1^O_2_), type II reaction.

After the absorption of photons the PS is transformed from its ground state to its triplet excited state via a short-lived excited singlet state (Figure 2) [11]. The triplet state can undergo two different reactions: (i) it can undergo electron or hydrogen atom transfer reactions with oxygen or a substrate producing free radicals and other reactive oxygen species; (ii) it can transfer its energy directly to ground-state triplet oxygen to form excited state singlet oxygen (Figure 3). The first process is called a type I reaction and the second a type II reaction [4]. The PDT effectiveness is therefore determined by oxygen supply and may be decreased in conditions where there is tissue hypoxia [12].

PDT as a treatment procedure has been accepted by the United States Food and Drug Administration for use in endo-bronchial and endo-esophageal cancer [13,14] and also as a treatment of premalignant and early malignant lesions of skin (actinic keratosis), bladder, breast, stomach and oral cavity [4,15].

The available body of literature suggests that there is clearly no single pathway that leads to cell death after PDT. In the present review we aim to describe and summarize different cell death pathways activated by PDT [16,17] and describe the key players controlling cell death related to PDT (Table 1).

## 2. PDT and Apoptosis

Apoptosis is a very complex, multi-step, multi-pathway cell-death program that is genetically encoded in every cell of the body [18]. It can be initiated either through the activation of death receptors or the mitochondrial release of cytochrome c [19]. Both events eventually lead to activation of caspase cascades known as ‘executioner caspases’ such as caspase-3, -6 and -7 [20,21]. The active executioner caspases cleave cellular substrates, which leads to characteristic biochemical and morphological changes observed in dying cells [22]. Cleavage of nuclear lamins is followed by chromatin condensation and nuclear shrinkage; cleavage of the inhibitor of the DNase CAD (caspase activated deoxyribonuclease) causes DNA fragmentation. Cleavage of cytoskeletal proteins leads to cell fragmentation and formation of apoptotic bodies [23]. The apoptotic process is tightly controlled by various proteins [24,25]. It is well known that resistance of tumor cells to apoptosis might be an essential feature of cancer development thus, modulation of the key elements of apoptosis signaling may directly influence therapy-induced tumor-cell death [26,27].

### 2.1. Involvement of Bcl-2 Family in PDT Response

In mammals the Bcl-2 family has at least 20 relatives, all of which share at least one conserved Bcl-2 homology domain. The family includes four other anti-apoptotic proteins: BclXL, Bcl-w, A1 and Mcll, and two groups of proteins that promote apoptosis: the Bax and the BH3-only families [28,29].

#### 2.1.1. The Pro-Survival Family

Bcl-2 and other members of the family potently inhibit apoptosis in response to many, but not all cytotoxic insults. Bcl-2 itself is required for the survival of kidney and melanocyte stem cells and mature lymphocytes [30], BclXL for neuronal and erythroid cells [31], and Bcl-w for sperm progenitors [32]. Bcl-2 and several other pro-survival molecules associate with the mitochondria outer membrane and the endoplasmic reticulum/nuclear membrane and maintain their integrity [33]. They can prevent cytochrome c release and subsequent caspase 9 activation. They probably also regulate the activation of several other caspases, independently of mitochondrial damage [34].

PDT is thought to induce photodamage of Bcl-2 and related antiapoptotic proteins and activate the proapoptotic members of the family [35]. Alteration in expression of members of Bcl-2 family proteins following PDT has been reported in various cell lines and tumors. Lutetium texaphyrin mediated PDT induced apoptosis in bovine retinal capillary endothelial (BRCE) cells involved changes in Bcl-2 and Bax levels while in human retinal pigment epithelial (RPE) cells changes in Bcl-x_L_ and Bak proteins were observed [36]. The PDT resistant HT29 human colon adenocarcinoma cell line showed variation in numerous genes and proteins including upregulation of Bcl-2 levels and downregulation of Bax [37]. Apoptotic death induced by 5-aminolevulinic (5-ALA) mediated PDT involved suppression of bcl-2 mRNA levels and elevation of Bax mRNA in cervical cancer cell line [38] and esophageal cancer cell [39]. This regimen also let to increase in Bax/Bcl-2 ratio in human malignant glioblastoma U87MG cells [40] while in U937 cells increase in Bak and Bax/Bcl-xL but decrease in Bid was observed [41]. 5ALA-PDT also caused a significant decrease in mRNA expression of Bcl-2 and increased the levels of Bax and Bad mRNA in cervical cancer in BALB/c nude mice [42]. Hypericin (HY) mediated PDT in human breast adenocarcinoma cell line involved downregulation of Bcl-xl and upregulation of Bax [43]. However, HY-PDT did not affect Bcl-2 levels in p53 null and wt-p53 expressing HCT 116 cells [44]. These studies indicated a possible involvement of the antiapoptotic Bcl-2 family members in PDT induced apoptotic cell death, but a more definite role for Bcl-2 comes from studies involving antisense treatment and overexpression approaches.

In the study by He *et al.* [45], employing a pair of Chinese hamster ovary cell lines that differed from one another by a transfected Bcl-2 gene, the ability of this gene to modulate PDT- induced apoptosis was investigated. PS used was a silicon phthalocyanine compound termed ‘Pc4’. Data from this research indicate that parental cells displayed a high incidence of apoptosis following PDT procedure, whereas Bcl-2-transfected cells exhibited a much lower incidence of apoptosis as assessed by DNA fragmentation by gel electrophoresis, flow cytometry and fluorescence microscopy. The clonogenic assays demonstrated that Bcl-2 was able to inhibit overall cell killing as well.

Kim *et al.* [46], however, reported a contradictory finding wherein overexpression of Bcl-2 in a subline of human breast epithelial cell MCF10A made them more sensitive to photodamage mediated by aluminum phthalocyanine. It was reasoned that transfection of Bcl-2 led to overexpression of both Bcl-2 and Bax. The mitochondrial photodamage resulted in selective degradation of Bcl-2 without affecting Bax, thereby increasing Bax: Bcl-2 ratio. A higher ratio is known to promote initiation of apoptosis [47], thus explaining the contradictory results. Similar results were obtained in a study by Srivastava *et al.* [48]. The two employed cell lines (RIF1 and A431) differed in their response to Pc4-PDT-induced apoptosis in antisense treatment and overexpression approaches. It was found that the overexpression of Bcl-2 in PDT apoptosis- sensitive human epidermoid carcinoma (A431) caused an enhanced apoptosis as well as increased Bax protein levels. Consequently, there was an increase in the Bax/Bcl-2 ratio that was associated with an increased apoptotic response. In the same study, the antisense Bcl-2 oligonucleotide was also used. Treatment of RIF1 cells with antisense oligonucleotide resulted in the significant reduction of their viability in the concentration-dependent manner as compared with cells treated with scrambled nucleotide. PDT treatment of apoptosis resistant RIF1 cells with antisense Bcl-2 oligonucleotide resulted in a significant induction of apoptosis 6 h following PDT. The antisense treatment was shown to result in enhanced caspase activity following PDT as compared to cells treated with PDT alone. The increase in caspase activity was maximal at 3 h post-PDT and declined at 6 h post-PDT. The team also observed that the antisense treatment resulted in significant PARP cleavage at 6 h post-PDT. The effect of antisense Bcl-2 oligonucleotide on the protein expression of Bcl-2 was assessed by immunoblot analysis and ELISA. Both assays demonstrated an almost 2-fold decrease in the protein expression of Bcl-2 in cells treated with antisense followed by PDT and a concentration-dependent decrease in the level of Bcl-2 with a concomitant increase in the extent of apoptosis. This decrease correlated with the increased sensitivity of RIF1 cells to PDT induced apoptosis. PDT of A431 cells, which were sensitive to PDT-mediated apoptosis, resulted in a significant time-dependent down-regulation of Bcl-2 protein. The overexpression approach of Bcl-2 resulted in an increase in apoptosis by PDT as compared to the normal wild type A431 cells. Moreover, the overexpression of Bcl-2 protein enhanced the PDT-mediated caspase activity indicating the increase in apoptosis.

This study documented that down-regulation of Bcl-2 with antisense oligonucleotide resulted in sensitization of PDT apoptosis-resistant cells to apoptosis; however, the overexpression of Bcl-2 in a PDT apoptosis-sensitive cell line resulted in the enhancement of the apoptotic response.

A similar approach was used in human gastric adenocarcinoma MGC803 cell line which was infected with antisense bcl-2 RNA retrovirus vector to study effect of 2-butylamino-2-demethoxy-hypocrellin A (2-BA-2-DMHA) photosensitization [49]. A significant reduction in Bcl-2 protein expression accompanied by an increased phototoxicity and susceptibility to apoptosis was observed.

Granvile *et al.* [50] in his overexpression model using benzoporphyrin derivative monoacid ring A (BPD-MA) and human acute myelogenous leukemia HL-60 cells observed that Bcl-2 overexpression may influence caspase 3 and caspase 6 activation and prevent the appearance of hypo-diploid DNA induced by PDT. However Bcl-2 overexpression was less effective at preventing cell death that occurred after photoactivation at high levels of PS.

A study performed by Kim *et al.* [51] showed that Bcl-2 transfection of MCF10A adhering human breast epithelial cell line resulted in a decrease in the light dose required for 90% loss of viability. Four hours after irradiation a substantial number of apoptotic cells were detected in the transfected subline, along with a markedly diminished intensity of MTO fluorescence. The authors concluded that a subline of MCF10A transfected with Bcl-2 was more sensitive to the lethal effects of PDT, showed a greater apoptotic response and a more rapid and extensive activation of caspase-3-like activity.

Another work indicating possible role of Bcl-2 in PDT response was reported by Xue *et al.* [52]. In this study it was shown that the anti-apoptotic protein Bcl-2 was highly sensitive to PDT mediated damage. It was observed that immediately following Pc4-PDT there was a marked reduction in the amount of Bcl-2 detected on the blot. Although there appeared to be a slow partial recovery of the Bcl-2 level in the cells 2 h post PDT, the control level was not restored. Overexposure of the same blot revealed the presence of a trace amount of a 23 kDa fragment in the treated cells. The loss of Bcl-2 was dose dependent and was observed for both endogenous and overexpressed Bcl-2 in several lines. The authors concluded that Bcl-2 was a target of PDT with Pc4, and PDT damage of the protein contributed to the efficient induction of apoptosis.

The study indicating that Bcl-2 may by a potential target in PDT, was also performed by Kessel *et al.* [53]. In this project by using 3 different photosensitizers classified as ‘mitochondrial’ (tin etiopurpurin, 9-capronyloxy-tetrakis porphyrin and meta-tetrahydroxyphenyl chloride) selective photodamage of Bcl-2 was observed, whereas the other family members were undamaged. This situation led to a substantial increase in Bax/Bcl-2 ratio, which has been described as a critical factor in PDT induced apoptosis.

This team provided additional evidence to the hypothesis that Bcl-2 photodamage can be a target for some photosensitizing agents by showing that the effects of the photosensitizer CPO can be mimicked by the Bcl-2 antagonist HA14-1 [54].

#### 2.1.2. The Pro-Apoptotic Family

The BH3-only proteins seem to act as damage sensors and direct antagonists of the pro-survival proteins, whereas the Bax like proteins act further downstream, probably in mitochondrial disruption. The BH3-only proteins are sentinels that can detect developmental death cues or intracellular damage. In healthy cells, they are restrained in diverse ways, including sequestration by the cytoskeleton. When unleashed by death signals, they switch off survival function by inserting their BH3 domain into a groove on their pro-survival relatives [55]. During apoptosis, Bax and Bak oligomerize in the mitochondria outer membrane and probably break its integrity, freeing pro-apoptotic proteins like cytochrome c, which allows the activation of caspase 9 [29].

The possible role of Bax in PDT-mediated apoptosis was showed by Srivastava *et al.* [56], when assessing the impact of PDT on the status of Bax protein in Bcl-2-overexpressing A431 cells. Using immunoblot analysis it was shown that PDT resulted in an increased level of Bax protein in these cells as compared with normal A431 cells and increase in Bax/Bcl-2 ratio.

The research performed by Usuda *et al.* [57] further supported the role of Bax in PDT-mediated cell death. The results indicated that Pc4-PDT caused the release of the Bax protein from the mitochondria of MCF7c3 cells but not from those of Bax-negative DU-145 cells. It was also observed that the apoptosis in Bax transfected cells was greater than in non-transfected cells.

Accumulating evidence suggests that PDT is an efficient inducer of apoptosis in many cancer cell lines, whereas the lack of the ability to undergo apoptosis does not protect cells from lethal effects of PDT. It therefore appears that the post-mitochondrial regulators, such as Bax protein, may be in the promotion of the late events of tumor responses. Thus, tumor cells with increased Bax/Bcl-2 ratio can be more effectively treated by PDT.

Bax was found to translocate to mitochondria during apoptosis in HeLa cells after PDT with zinc (II) phthalocyanine [58]. Npe6 mediated PDT involved Bax protein as indicated by a study in Lewis lung carcinoma cells transfected with IL-6 that have higher Bax protein levels than the parent cells. Bax was also induced remarkably by NPe6 mediated PDT in the transfected cells. Moreover the decreases in the expression of Bcl-2 after PDT in LLC and LLC-IL-6 cells were similar thereby eliminating modulation of Bcl-2 expression levels by PDT as an explanation in this case [59]. The dependence of Pc4-PDT induced apoptosis on Bax remains under active investigation and reports from Chiu *et al.* [60,61] indicated that commitment to cell death after PDT occurs prior to Bax activation while Usuada *et al.* [62] reported that Smac/DIABLO promotes apoptosis after Pc4-PDT in a Bax dependent manner. Furthermore, Bax was not found to be essential for Photofrin-PDT induced apoptosis in human lung adenocarcinoma cells (ASTC-a-1) [63]. In the absence of Bax/Bak, autophagy appears to be predominantly responsible for cell death following PDT with different photosensitizers [64-66].

## 3. Cytochrome c Release after PDT

It is generally recognized that mitochondria play a critical role in the apoptotic cascade by controlling the release of crucial factors involved in that process [67]. Among these factors cytochrome c plays an essential role. During the process of apoptosis, cytochrome c is released from mitochondria into the cytosol. Release of cytochrome c from mitochondria is controlled by proteins of the Bcl-2 family. In the cytosol, cytochrome c activates the caspases—a family of killer proteases—through formation of a complex with Apaf-1 (for apoptotic-protease activating factor-1), procaspase-9 and ATP or dATP. [68]. The opening of the mitochondrial membrane permeability transition pores, which results in the dissipation of the mitochondrial membrane potential (Δψ_m_), has been proposed as the main mechanism for release of cytochrome c. Chiu and Olenick [69], showed that treatment of LY-R cells with Pc4 based PDT resulted in release of cytochrome c to the cytoplasm after 15 min, as estimated by an immunohistochemical method, and the loss of Δψ_m_ depended on PDT dose and the post treatment time. The observed loss of Δψ_m_ was only in those cells receiving the highest dose of PDT. It was concluded that the release of cytochrome c from mitochondria is independent of the loss of Δψ_m_ action after Pc4 mediated PDT.

Another work indicating an important role of cytochrome c in PDT-induced apoptosis was carried out by Vantieghem *et al.* [70]. In his approach, the overexpression of Bcl-2 in Pc60R1R2 cells revealed that mechanism of cytochrome c release after hypericin mediated PDT is caspase-dependent. The observed overexpression of Bcl-2 remarkably delayed cytochrome c release, but it did not protected cells from PDT induced death. These results showed that in cells overexpressing Bcl-2 addition of zVAD-fmk significantly suppressed cytochrome c release and apoptosis.

Another support for the hypothesis that cytochrome c plays a vital role in PDT-induced apoptosis was provided by Varnes *et al.* [71]. In this study, an LD_99.9_ dose of Pc4 PDT induced loss of cytochrome c from the mitochondria of LY-R cells, but also inhibited respiration and caused activation of caspase 3-like proteases. All events took place within 15 min of light exposure. This study provided clear evidence that cytochrome c leakage is dependent on activation of proteolytic proteases after PDT.

## 4. Involvement of Death Receptors in PDT Response

All death receptors belong to TNFR superfamily (tumor necrosis factor receptor) [72]. Most of them act as transmembrane signal transducers that respond to ligand binding. Some of them, however, do not transduce signals, but rather function as decoy receptors that compete for the interaction of cognate ligands with their signaling receptors. Each member of the TNFR superfamily has at least one specific ligand, but there are other ligands that bind to several receptors. The most complex example is APO2/TRAIL, which binds five different receptors [73].

TNFR signaling was discovered to be an important factor in immune response and such members like Fas and APO2/TRAIL induce apoptosis through a p-53 independent mechanism. The signaling members of TNFR superfamily can be divided into two main subgroups. One class contains cytoplasmic death domain, whereas the other class does not. Death domains mediate interaction of death receptors with death-domain–containing adaptor proteins and then act in the caspase-dependent pathway. Death domain containing receptors activate the apoptotic pathway by caspase-8 mediated cleavage of the pro-apoptotic Bcl-2 superfamily member BID [74]. This protein interacts with other molecules like Bax and Bak, which cause release of mitochondrial cytochrome c and SMAC/DIABLO, activating caspase-9 and eventually caspase-3. The other group use TNFR-associated factor to link these receptors to serine/threonine protein kinase cascades that regulate gene transcription [75].

Because treatment with factors that activate death receptor signaling in cancer cells may be an effective anticancer strategy it has been investigated whether PDT can affect this pro-apoptotic pathway.

The early evidence comes from the study designed by Ahmad *et al.* [76] where the involvement of the cell surface receptor Fas (CD95) pathway in A431 cells in Pc4 mediated PDT was investigated. It was observed that a significant time-dependent increase in the protein expression of Fas at 5, 15, 30 and 60 min post PDT occurred. In an immunoblot, Fas protein was observed as a doublet, which may indicate the insoluble and soluble forms of Fas. By using immunoprecipitation it was determined that Pc4-PDT resulted in a multimerization of Fas protein leading to its activation. To confirm that PDT induced apoptosis is due to Fas activation the involvement of Fas associated death domain level (FADD) was examined by using immunoblot analysis. It confirmed a time-dependent increase, for up to 1 h post-PDT in Fas protein levels. Pc4-PDT also caused activation of FADD-like interleukin-1 beta-converting enzyme (FLICE), which was evident from the appearance of cleaved products of pro-casapase-8.

To further evaluate the role of Fas in PDT mediated apoptosis, Ali *et al.* [77] used a model of Hypocrellin A (HA) and Hypocrellin B (HB) as photosensitizers in CNE2 and TW0-1 cells. This group observed elevated levels both Fas and FasL in both examined cell lines and these higher levels coincided with increase in phototoxicity of PDT. The enhanced expression of CD95/CD95L was detectable within 2 h after irradiation using HA and HB and was significantly increased at 3 h following light exposure. The specific involvement of Fas/FasL system in CNE2 and TW0-1 cell death induced by HA and HB was further supported by significant inhibition of apoptosis in the presence of either anti-CD95 or anti-CD95L antibodies.

There is also strong evidence that APO2/TRAIL is involved in PDT mediated apoptosis. Schempp *et al.* [78] used Hypericin mediated PDT in Jurkat cells and by using neutralizing antibodies against Fas, FasL, TRAIL and TNFR1 discovered that post PDT apoptosis can be inhibited by polyclonal anti-TRAIL antibody. The specificity of the inhibitory activity of this antibody was demonstrated by blocking TRAIL mediated killing of Jurkat cells. However, the mechanism of Hypericin-PDT mediated apoptosis based on TRAIL/TRAIL-receptors involvement is not clear and it may include increased shedding of TRAIL, stabilization of death inducing TRAIL receptors or down regulation of decoy receptors.

Further support for this hypothesis was provided by Granville *et al.* [79], who described a two-fold increase in the number of cells containing hypo diploid DNA after combined therapy PDT + TRAIL. They also described that combined therapy of PDT and Fas-ligand increased the number of apoptotic cells seven times. The final conclusion was that Jurkat cells undergoing PDT procedure become more sensitive to Fas-ligand and TRAIL induced killing.

### 4.1. NF-κB Involvement in PDT-Mediated Apoptosis

Nuclear factor kappa B (NF-κB) is a term referring to a group of dimeric transcription factors that belong to the Rel family and are regulated via shutting from the cytoplasm to the nucleus in response to cell stimulation [80]. The Rel proteins belong to two classes. The first class includes RelA, RelB and c-Rel, proteins that are synthesized as mature products and do not require proteolytic processing. The second group is encoded by the Nfkb1 and Nfkb2 genes, whose proteins are first synthesized as large precursors that require proteolytic processing to produce mature forms. NF-κB dimers are held in the cytoplasm through specific inhibitors of kappa B, the IκBs [81]. IkB members undergo rapid ubiquitin-dependent degradation after exposure to a variety of agonists, which activate the IκB kinase complex (IKK) [82]. This complex induces the phosphorylation-dependent removal of the IKB-like *C*-terminal domain of NF-κB, which allows dimers to translocate to the nucleus. Once in the nucleus the dimers target genes that belong to four broad categories: immunoregulatory and inflammatory genes; anti-apoptotic genes; genes that positively regulate cell proliferation; genes that encode negative regulators of NF-κB. NF-κB is also known as an inhibitor of programmed cell death [83]. This factor activates transcription of several genes that are known to block the induction of apoptosis by TNF superfamily members [84]. The anti-apoptotic factors that are induced by NF-κB include cellular inhibitors of apoptosis (cIAPs), FLICE and members of Bcl-2 family. NF-κB can also attenuate the apoptotic response to genotoxic anticancer drugs and radiation therapy [84].

PDT produces an oxidative stress that can result in activation and translocation of NF-κB to the nucleus, as originally shown for treatment of L1210 murine leukemia cells with Photofrin and light. [85]. The claim that NF-κB may be a target for PDT has been convincingly demonstrated by Granville *et al.* [86]. The photodynamic treatment of HL-60 cells with Verteporfin has no detectable effect on cellular IκB levels by 1 h. Furthermore, lysates prepared up to 3 h post PDT did not contain detectable IκB degradation products. However, the nuclear lysates revealed low protein levels showing reliable assessment of κB binding activity. After the PDT procedure, HL-60 cells transfected with a Luc reporter gene exhibited increased luciferase activity, confirming that photosensitization leads to productive NF-κB-mediated gene transcription. The mechanism by which PDT induce NF-κB translocation to the nucleus is so far unclear, but this study confirmed that NF-κB provides an anti-apoptotic signal for cells exposed to PDT.

## 5. Mitogen-Activated Protein Kinases (MAPK) Involvement in PDT-Mediated Apoptosis

Mitogen-activated protein kinases are proline-directed Ser/Thr protein kinases activated by dual phosphorylation on both tyrosine and threonine residues [87]. These enzymes are critical components of a complex cellular signaling network that ultimately regulates gene expression in response to variety extracellular stimuli. The three well known MAPK families are: the extracellular signal-regulated kinases (ERKs), the c-Jun N-terminal kinases/stress-activated protein kinases (JNKs/SAPKs), and the p38 MAPK [88]. Each of these enzymes is a target for phosphorylation cascades in which the sequential activation of three kinases constitutes a common signaling pathway. The best characterized MAPK pathway is the Ras/Raf/MEK cascade leading to the activation of ERK1/2 in response to growth factors [89]. JNK and p38 are key mediators of stress signals and inflammatory response [90].

A link between SAPK and p38 pathways and apoptosis has been suggested in several studies [91,92]. The evidence of the involvement of JNK1 and p38 was provided by Assefa *et al.* [93]. In hypericin-mediated PDT of A431, HaCaT, HeLa and L929 cell lines theye observed a rapid and persistent activation of JNK1 and severely inhibition of the basal levels of ERK2. It was also demonstrated that photo-activated hypericin blocked the EGF-mediated activation of ERK2, and the inhibition of ERK2 pathway was irreversible. They also observed that JNK1 activation occurred independently from caspase activities. Using simultaneous inhibition of both stress kinases they observed significant sensitization of cells to PDT-hypericin mediated apoptosis. They concluded that both JNK1 and p38 MAPK pathways played an important role in cellular resistance to PDT-induced apoptosis with hypericin.

The involvement of stress kinases in PDT mediated cell death was also investigated by Chan *et al.* [94]. They evaluated the role of JNK1 and involvement of singlet oxygen in triggering the JNK pathway. PDT triggered activation of stress kinase pathway in two steps: firstly the formation of singlet oxygen induced rapid activation of JNK, which led to activation of the caspase cascade and apoptosis. Secondly activated caspase-3 could then act on several apoptotic substrates including PAK2. Cleavage of PAK2 released its C-terminal catalytic fragment as a 36 kDa active kinase that can further perform a second step in JNK activation.

Xue *et al.* [95] investigated the involvement of Etk/Bmx kinase in PDT mediated apoptosis. Etk/Bmx is a newly discovered tyrosine kinase, commonly expressed in prostate epithelial and carcinoma cells. It might play an important role in the growth, development and anti-apoptotic action in epithelial cells. Using an overexpression model in LNcaP cells they found that Etk/Bmx is an effector of PI3-kinase and that the PI3-kinase/Etk pathway is involved in the protection of prostate carcinoma cells from apoptosis in response to PDT.

## 6. Involvement of Other Factors in PDT Mediated Apoptosis

Ceramide is implicated in the cell-signaling pathway involved in apoptosis as it acts as a second messenger to permeabilize the outer mitochondrial membrane and facilitate the release of cytochrome c from the mitochondria [96]. Other modes of ceramide-mediated cell death involve the activation of stress-activated protein/JUN kinase (SAPK/JNK) [97], dephosphorylation of the retinoblastoma protein and upregulation of transcription factors. Evidence of ceramide involvement in PDT-mediated apoptosis was provided by Separovic *et al.* [98]. In phthalocyanine PDT model they demonstrated in the U937 cell line elevated levels of ceramide by 45, 37, 67, 118 and 134% at 1, 10, 30, 60 and 120 min, respectively, after PDT procedure. In CHO cells ceramide generation in response to PDT was rapid but did not reach such a high level. Ceramide accumulation was also observed in RIF1 cells. In this model ceramide levels were not different from control level for the first 10 min post-PDT but then reached a maximum of 118%, maintaining this level up to 2 h. The PDT dose produced a 99% loss of clonogenicity, however in RIF1 cell there was no evidence of apoptosis.

p21/WAF1 involvement in PDT was investigated by Ahmad *et al.* [99]. They found that cells subjected to PDT resulted in a growth arrest particularly observed in G_0_-G_1_ phase. The treatment caused an accumulation of 47%, 51% and 69% growth-arrested cells at 3, 6 and 12 h post-PDT, respectively. They further assessed the influence of PDT on WAF1/p21 induction and using Western blot analysis revealed a significant induction of WAF1/p21 at 3, 6 and 12 h after treatment, when compared with basal levels. In addition, PDT resulted in a time-dependent decrease in cyclin D1, cyclin E, cdk2 and cdk6. The decrease of cyclin D1 and cdk6 was markedly more pronounced than that of cyclin E and cdk2. To confirm this data they investigated the effect of PDT on kinase activities associated with cdk. The radioactive kinase activity assay showed that PDT almost universally resulted in a time-dependent decrease in kinase activities associated with all the cdks and cyclin examined. They also examined the effect of PDT on the binding between WAF1/p21-cyklin and WAF1/p21-cdk and also cyclin-cdk and found that PDT resulted in increased binding of cyclin D1 and cdk6 toward WAF1/p21, whereas the binding of cyclin E and cdk2 did not change.

## 7. PDT and Necrosis

Necrotic cell death has been described as a violent and quick form of degeneration affecting large fractions of cell populations, characterized by cytoplasmic swelling, destruction of organelles and disruption of the plasma membrane, leading to the release of intracellular contents and consequent inflammation [100].

Necrosis has been referred to as accidental cell death, caused by physical or chemical damage and has generally been considered an unprogrammed process [101]. It is characterized by a pyknotic nucleus, cytoplasmic swelling, and progressive disintegration of cytoplasmic membranes, all of which lead to cellular fragmentation and release of material into the extracellular compartment. In necrosis, decomposition is principally mediated by proteolytic activity, but the precise identities of proteases and their substrates are poorly defined [102].

Studying the factors and parameters that cause cellular necrosis after PDT is not as easy as studying those factors which lead to apoptosis. The crucial factors in determining the type of cell death, e.g., apoptosis or necrosis following PDT are: the cell type, the presence of an intact set of apoptosis machinery, the subcellular localization of the PS, the light dose applied to activate it locally, and the oxygen partial pressure [103]. One factor that can be agreed upon by all commentators is that high dose PDT (either a high photosensitizer concentration or a high light fluence or both) tends to cause cell death by necrosis, while PDT administered at lower doses tend to predispose cells towards apoptotic cell death.

Nagata and colleagues [104] used the amphiphilic PS ATX-S10 (Na) and human malignant melanoma cells and found that light doses that led to less than 70% cytotoxicity induced mainly apoptosis; by contrast, most cells appeared necrotic with doses that induced 99% cytotoxicity. A common feature of the apoptotic program initiated by PDT is the rapid release of mitochondrial cytochrome c into the cytosol followed by activation of the apoptosome and procaspase-3. With PS localized in the plasma membrane the photosensitization process can rapidly switch the balance towards necrotic cell death likely due to loss of plasma membrane integrity and rapid depletion of intracellular ATP [105]. It is also possible that high doses of PDT can photochemically inactivate essential enzymes and other components of the apoptotic cascade such as caspases. For instance, Lavie and coworkers [106] used the perylenequinones (hypericin and dimethyl tetrahydroxyhelianthrone) and found high dose PDT inhibited apoptosis by interfering with lamin phosphorylation, or by photodynamic cross-linking of lamins.

Xue and Oleinick [107] compared Pc4-mediated PDT of MCF7 cells that lack caspase 3 with the same cell line with caspase 3 transfected back in. They found apoptotic indicators only in the caspase expressing cells which also showed more loss of viability by an assay involving reduction of a tetrazolium dye; however both cell lines showed an equal degree of cytotoxicity by a clonogenic assay. Dahle, Steele and Moan reported [108] that the mode of cell death induced by PDT depended on cell density. They seeded Madison Darby canine kidney II cells (MDCK II) at two different densities and incubated them with meso-tetra(4-sulfonatophenyl)porphine (TPPS4) for 18 h, washed and irradiated with blue light. Four hours later the cells were studied by fluorescence microscopy. With <55% total cell death the apoptotic fraction was significantly higher for cells in confluent monolayers than for cells growing in microcolonies at equitoxic doses. Confluent cells were 2.9 times more sensitive than cells in microcolonies partly due to a 1.5 times higher uptake of TPPS4 in monolayer cells. The difference in mode of cell death for the different cell densities was not related to any observable difference in subcellular localization pattern of TPPS4 at equitoxic doses of PDT.

## 8. PDT and Autophagy

Autophagy is a catabolic cellular mechanism that allows the cell to maintain a balance between the synthesis, degradation, and recycling of cellular products [109]. A variety of autophagic processes exist, all of which involve the lysosomal degradation of the cellular organelles and proteins. The most well-known mechanism proceeds in the following manner: A double membrane structure called autophagosome surrounds the target region, creating a vesicle that separates its contents from the rest of the cytoplasm. This vesicle is then transported and fused to the lysosome, forming a structure called the autophagolysosome, the contents of which are subsequently degraded by lysosomal hydrolases [110]. Besides facilitating the disposal of unwanted proteins, organelles, and invading microorganisms, autophagy also allows a cell to reallocate its nutrients from unnecessary processes to life-essential ones in times of starvation or stress.

The term “autophagy” originated in 1963 at the Ciba Foundation Symposium on Lysosomes, where it was used to describe the presence of membrane vesicles that contain disintegrating organelles and cytoplasm components. Evidence soon confirmed that autophagy is an adaptive, energy-generating process. Molecular control of autophagy came into focus in the late 1990s. Signaling pathways governing this process became better understood after the identification of the target of rapamycin kinase (TOR), which controls cell growth and protein synthesis [111]. In 1997, Ohsumi's group showed that yeast autophagy is similar to that of mammals, which allowed them to analyze the process in the genetically tractable yeast system. This study led to the discovery of the first autophagy-related gene, ATG1. Shortly thereafter, the first mammalian autophagy genes were identified [112].

One of the first diseases associated with autophagy was cancer [113]. It was discovered that Beclin1, an essential protein for autophagy, is also a tumor suppressor [114]. Further studies on the role of autophagy in cancer revealed some interesting properties of the disease. Initially, autophagy suppresses tumor growth through its production of Beclin1. This changes as the tumor becomes more advanced; autophagy begins to promote tumor progression by providing the mechanism for cells in the central, low nutrient, part of the tumor to obtain the energy that they need to stay alive. It was also found that autophagy blocks apoptotic pathways, thereby protecting cancer cells from treatment [114]. On the other hand, some cancer therapies induce autophagic cell death of tumor cells. This two-sided effect of autophagy on tumors can be exploited by anticancer therapy to provide better treatment for cancer patients.

It is still unclear exactly how autophagy affects the outcome of PDT [66,115,116]. In general, mammalian cells use autophagy as a defense against ROS mediated damage by clearing the cell of damaged organelles [117]. Depending on the type of ROS and degree of oxidative injury, PDT may stimulate autophagy [118] that either acts in a cytoprotective manner or induces autophagic cell death [119]. Autophagy may play a role in PDT induced apoptosis, but the two processes can also occur independently of one another [120]. A study done on murine leukemia L1210 cells found that a wave of autophagy occurs right before apoptosis [121]. It was also found that prevention of autophagy by silencing the Agt7 gene allowed photo-killing to occur at lower light doses. This observation is consistent with the theory that autophagy is a defense mechanism against PDT induced ROS [122]. The situation is different for tumor cells that do not have the ability to undergo apoptosis through a deficiency in Bax and Bac, which regulate the apoptotic pathway. In these cells, PDT induced autophagy stimulates a necrotic, caspase independent cell death [64]. Suppression of autophagy in apoptosis-deficient cells resulted in the inhibition of cell death during PDT. In general, the induction of autophagy in PDT treated cells occurs independently of an apoptotic outcome. While autophagy seems to play a pro-survival role in tumor cells that are capable of apoptosis, it has been shown to promote death in cells that are apoptosis-deficient.

In order to understand how PDT affects autophagy it is important to take note of the PDT affected proteins that are involved in this mechanism. Many proteins, some directly involved in the autophagic process, are damaged by PDT induced ROS. Some ER and mitochondrial photosensitizers cause damage to Bcl-2, to which Beclin1 (a pro-autophagic protein) binds. The IP3 protein, a regulator of the autophagic process associated with the ER [123,124] is affected by phthalocyanine photosensitizer Pc 4 [125]. The photosensitizer AlPcS causes damage to the mammalian target of rapamycin, mTOR, a cell growth regulator that takes part in the autophagic signaling pathway [126]. Other proteins, like Beclin1, Atg5, and Atg7, appear to be unaffected by PDT. Although many proteins involved in the autophagic process are photodamaged by PDT, it appears that those involved in the formation of autophagosomes remain active [120].

PDT can also affect autophagy by damaging organelles that are directly involved in the process. Several photosensitizers target autophagy-related organelles such as lysosomes and endosomes. When tetra(4-sulphonatophenyl)-porphine (TPPS4) photosensitizer is used photo-oxidation of the organelle matrix occurs [127]. In this type of PDT, lysosomal enzymes are inactivated before the membrane ruptures, which allows for specific targeting of this organelle without causing damage to the rest of the cell. This treatment can be used to selectively enrich autophagosomes [128]. On the other hand, NPe6 and TPPS2 photosensitizers bind to the lysosomal membrane, causing it to rupture upon irradiation. The released, but not neutralized proteases can cause induction of apoptosis by cathepsin-mediated cleavage of Bid [129]. The lysosomes, autophagosomes, endosomes, and autolysosomes of the cells treated with this amphiphilic photosensitizer will have it in their membranes. Upon irradiation, the membranes will break and release their contents into the cytoplasm. Based on these results, it can be concluded that the effect of PDT on the autophagy of a tumor cell depends on the type of photosensitizer used.

The effects of a damaged lysosome on the autophagic process were explored in an autophagic flux study of murine hepatoma 1c1c7 cultures. These cultures were sensitized with NPe6 and irradiated with LD90 light dose. Acridine orange (AO) staining of acidic organelles was done in order to observe the effect of PDT on the lysosome. No lysosomal damage was observed when cells were exposed to either light or photosensitizer alone. In those cells that received PDT, AO staining was completely lost within an hour, and within two hours autophagosome accumulation was observed [130]. This indicates that autophagosomes can be formed in the absence of lysosomes, but autophagy cannot be completed due to the lack of these organelles.

Based on studies with various cancer lines and photosensitizers it can be concluded that PDT directly induces autophagy. This is independent of photosensitizer target, as autophagy was observed with photosensitizers that localize in the ER, mitochondria, lysosomes, and endosomes. A second conclusion that can be drawn is that apoptosis often occurs in cells that are already undergoing autophagy and is also a result of PDT. The rates of autophagy and apoptosis depend on the cancer cell type, photosensitizer, and light dosage. Depending on cell type, autophagy will either promote or prevent cell death from PDT. In cells that are able to undergo apoptosis autophagy alleviates the destructive effects of PDT by recycling damaged organelles [131]. This opens up a possibility that PDT of these cancer cells can be enhanced by suppressing pro-autophagic proteins. Autophagy has the opposite effect on cells that are apoptosis-deficient, promoting cell death through necrosis. The last conclusion that can be made is that autophagic processes are compromised, but not prevented, in PDT protocols that employ lysosome targeting photosensitizers. In these procedures, autophagy is initiated by formation of autophagosomes but cannot be completed by fusing with the lysosome. This is because the proteins involved in autophagosome formation (Beclin1, Atg5) are not photodamaged by PDT.

## 9. Conclusions

**Figure 4.**
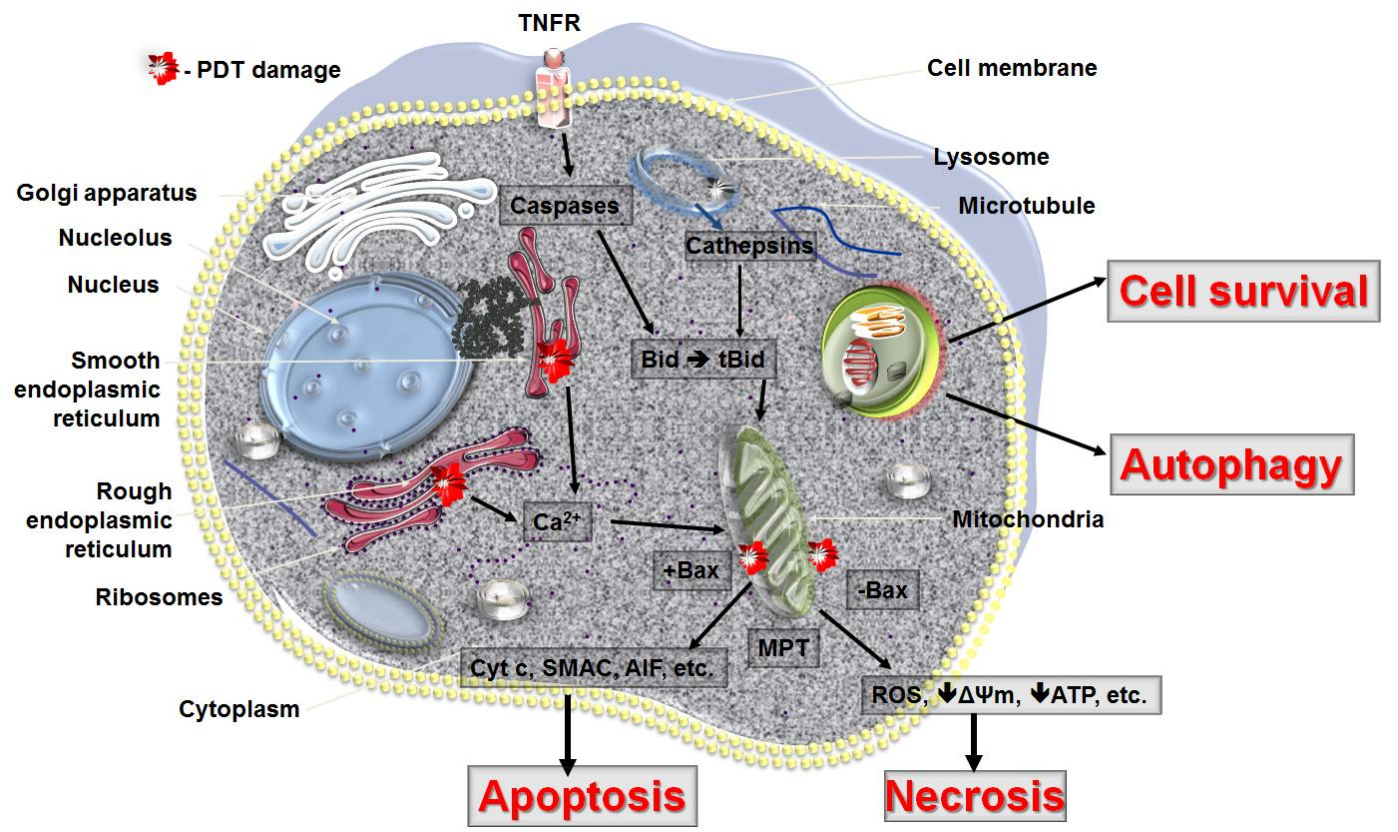
Cell death pathways in PDT. The mode of cell death observed after PDT to some extent depends on the intracellular localization of the PS and PDT related damage to that organelle. PDT with PS localizing in mitochondria will lead to loss of membrane permeability and release of pro-apoptotic mediators while ER damage will release cellular deposits of calcium. PS that accumulates in lysosomes will release proteolytic enzymes upon illumination. Lysosomes may also fuse with autophagosomes to hydrolyse damaged organelles and recycle them during autophagy. In the excess of damage the cell will not survive despite initiation of autophagy. Necrosis as well as autophagy may be a dominant cell death mode after PDT when apoptosis is dysfunctional. It should be remembered that several PS may localize in more than one organelle and the activation of cell death pathways may occur concurrently (adapted from Oleinick *et al.* [132]).

PDT can lead to all three forms of cell death, namely apoptosis, necrosis and autophagy (Figure 4). The response to PDT may vary not only with the cell type or its genetic or metabolic potential but also with the experimental model, total fluence delivered, different types of photosensitizers and their intracellular localization. The initial site of PDT-related damage may determine which cell death pathway is initially activated. The extent of PDT related damage may also regulate how the PDT treated cells respond. It is possible that the autophagy process is activated as an initial rescue mechanism, when the PDT damaged cells try to contain and removed damaged proteins. Only when the PDT damage is sufficiently robust and the cells are damaged beyond repair does apoptosis occur. PDT, at its highest dose may also lead to necrosis, as the proteins that participate in both autophagy and apoptosis may be immediately destroyed and the cellular integrity may be broken.

It is also important to bear in mind that most of the reported *in vitro* data are also difficult to translate into the *in vivo* situation where unequal light distribution or inhomogeneous photosensitizer accumulation may lead to a variable cellular response within PDT treated tumors. Additionally, the shutdown of tumor vessels may lead to local depletion of nutrients and oxygen and therefore trigger secondary PDT related necrosis. Lastly, as PDT leads to activation of tumor directed immune response, some cancer cells are killed via apoptosis by cytotoxic T cells.

The understanding of the cascade of events playing a role in PDT-mediated apoptosis is far from complete. Defining these events will hopefully result in designing better PDT protocols, which could have wider applications as a cancer therapeutic modality.

## Figures and Tables

**Table 1.** Major cell death mechanism activated by Photodynamic therapy (PDT).

**Anti-tumor PDT mechanisms**		
**Direct cell damage**	**Organelles**	**Processes**
	**Mitochondria:** Cytochrome c release Bcl-2 damage	**Apoptosis**
	**Cytoplasm:** NFκB damage	
	**Endoplasmatic reticulum:** Beclin 1, mTOR activation	**Autophagy**
	**Cell membrane disintegration**	**Necrosis**
**Vascular shutdown**	**Local depletion of oxygen and nutrients**	**Apoptosis** **Necrosis** **Autophagy**
**Activation of immune response**	**Cytotoxic T cells**	**Granzyme mediated apoptosis**
